# Serotonin Syndrome after PACU Administration of Tramadol and Meperidine

**DOI:** 10.5152/TJAR.2022.21355

**Published:** 2022-08-01

**Authors:** Rachel Gray, Arnold Moore, Fiona Berry, Farzana Afroze, Divya Cherukupalli

**Affiliations:** 1 Department of Anaesthesiology, Albany Medical Center, New York, USA; 2 Albany Medical College, New York, USA

**Keywords:** Enhanced recovery after surgery, meperidine, perioperative period, serotonin syndrome, tramadol

## Abstract

Serotonin syndrome, malignant hyperthermia, and neuroleptic malignant syndrome are life-threatening conditions with similar clinical presentations, all of which may occur in post-operative patients. The rarity of these conditions has limited their research as well as the ability to recognise and treat them effectively. We present the case of a 61-year-old male who developed altered mental status, respiratory distress, fever, and haemodynamic instability following post-operative administration of tramadol and meperidine. The differential diagnosis process and process of elimination were used to diagnose this patient with serotonin syndrome. Chart review was used to review the details of the case to write this report. Serotonin syndrome was eventually diagnosed in the context of the symptoms following the administration of 2 serotonergic agents. The patient’s symptoms improved with supportive care and did not recur. This case is one of the first published reports of serotonin syndrome resulting from an interaction between tramadol and meperidine, highlighting the importance of constant vigilance in the perioperative period when serotonergic agents are involved. The increased usage of serotonergic antidepressants and Enhanced Recovery After Surgery protocols calling for serotonergic analgesics represent a significant and underrecognised potential for serotonergic multidrug interactions to occur.

**Main Points**

- Serotonin syndrome (SS), malignant hyperthermia, and neuroleptic malignant syndrome are life-threatening conditions with similar clinical presentations, all of which may occur in post-operative patients.
- The increased usage of Enhanced Recovery After Surgery protocols calling for serotonergic analgesics and the high rate of serotonergic antidepressant prescriptions represent a significant and underrecognised potential for serotonergic multidrug interactions to occur.
- This case is one of the first published reports of SS resulting from an interaction between tramadol and meperidine, highlighting the importance of constant vigilance in the perioperative period when serotonergic agents are involved.

## Introduction

Serotonin syndrome (SS), malignant hyperthermia (MH), and neuroleptic malignant syndrome (NMS) are life-threatening conditions with similar clinical presentations that arise in response to exposure to certain drugs. Rare occurrences of these conditions have limited their research and the ability to recognise and treat them effectively. This report will present the case of a post-operative patient in the post anaesthesia care unit (PACU) who developed SS secondary to the administration of tramadol and meperidine. As Enhanced Recovery After Surgery (ERAS) protocols become more prevalent and antidepressant prescription rates rise, it has become more important than ever for a timely diagnosis of SS. A written informed consent was obtained from the patient.

## Case Presentation

A 61-year-old male with an American Society of Anesthesiologists physical status II and a history of Crohn’s disease presented for an exploratory laparotomy and repair of enterocutaneous fistula. He was haemodynamically stable intraoperatively. He was placed on an ERAS Protocol which entailed multimodal analgesia and the placement of bilateral transverse abdominal plane (TAP) catheter blocks for recovery.

In the PACU, he received fentanyl, cyclobenzaprine, and tramadol. He began to develop chills and was prescribed low-dose meperidine. He then developed diffuse erythema, hypotension, and respiratory distress. Transverse abdominal plane catheters were held. Given concern for anaphylaxis, his airway and skin were inspected. No oedema or urticaria were present. Lung examination revealed bilateral wheeze, which was treated successfully with supplemental oxygen and sequential ipratropium bromide—albuterol sulfate nebulisers. He was also given diphenhydramine. His hypotension resolved with a phenylephrine infusion, which was discontinued within 10 minutes of initiation. He subsequently developed a fever, with a maximum temperature of 40˚C, rigors, altered mentation, hypertension, and tachycardia. Given the change in his status, there was a concern for SS versus MH versus septic shock. He was cooled with a cooling blanket and ice packs and was given IV acetaminophen. Cyproheptadine was ordered to be available at bedside; however, his symptoms gradually improved with supportive care. An A-line was placed, and an arterial blood gas, blood cultures, chemistry, and complete blood count were sent for analysis. Urine output was monitored via a Foley catheter, and once stable, the patient was admitted to the intensive care unit for further monitoring. Blood cultures were negative, and all other blood work was within normal limits.

## Discussion

Serotonin syndrome, MH, and NMS are life-threatening conditions that require prompt diagnosis and treatment. However, differentiating between them can be challenging as they all present with an altered mental status that progresses to neuromuscular symptoms, autonomic dysregulation, and hyperthermia.

**Figure 1.**
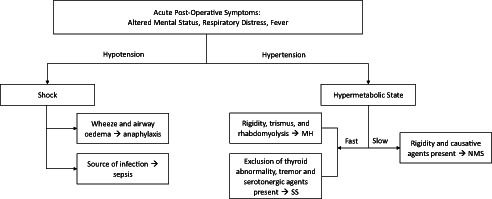
Flow diagram outlining a potential diagnostic process for post-operative symptoms of altered mental status, respiratory distress, and fever.

The patient in our case first presented with hypotension, erythema, and respiratory distress. These symptoms pointed to a differential diagnosis of anaphylaxis, SS, MH, NMS, and septic shock (Figure 1). In our case, vasodilatory processes including anaphylaxis and septic shock were eliminated as the patient developed hypertension. Anaphylaxis became less likely as the patient did not develop urticaria or facial oedema.^1^ Sepsis was conclusively eliminated when blood cultures were negative.^2^ Neuroleptic malignant syndrome was eliminated as the patient was not on any known causative agents and had a rapid onset of symptoms.^3^ Finally, the patient did not develop classic signs of MH, including muscle rigidity, trismus, rhabdomyolysis, and subsequent kidney failure.^4^ Serotonin syndrome was concluded as the diagnosis through this process of elimination. While the patient’s initial hypotension was not consistent with the typical clinical presentation of SS, serotonin is known to cause vasodilation.^5^ Further, the disease process was self-limiting once the serotonergic agents were removed.

There are many factors that put patients at an increased risk of SS given current trends in anaesthesiology. Tramadol has grown increasingly popular as ERAS protocols calling for opioid-sparing analgesia become more common. Additionally, antidepressant prescription rates have increased in the past 2 decades, and patients may be less likely to report antidepressant use than use of other medications.^6,7^ The increased implementation of ERAS protocols and the increased antidepressant prescription rates may contribute to a continual increase in the use of serotonergic agents, leading to more potential circumstances for SS to occur. This is one of the first reports of an interaction between tramadol and meperidine resulting in SS, highlighting the importance of vigilance regarding SS and other similarly presenting diseases in the perioperative period.

## Conclusion

This is the first reported case of SS induced by a tramadol and meperidine interaction. As ERAS protocols with tramadol grow in popularity and increasing numbers of patients are prescribed antidepressants, it is imperative for providers to watch for SS in the perioperative period and be aware that patients may not report the selective serotonin reuptake inhibitors (SSRIs) they are taking.

## References

[1] Webb LM Lieberman P . Anaphylaxis: a review of 601 cases. Ann Allergy Asthma Immunol. 2006;97(1):39 43. 10.1016/S1081-1206(10)61367-1) 16892779

[2] Murray PR Masur H . Current approaches to the diagnosis of bacterial and fungal bloodstream infections in the intensive care unit. Crit Care Med. 2012;40(12):3277 3282. 10.1097/CCM.0b013e318270e771) 23034460 PMC4201853

[3] Ware MR Feller DB Hall KL . Neuroleptic malignant syndrome: diagnosis and management. Prim Care Companion CNS Disord. 2018;20(1). 10.4088/PCC.17r02185) 29325237

[4] Rosenberg H Pollock N Schiemann A Bulger T Stowell K . Malignant hyperthermia: a review. Orphanet J Rare Dis. 2015;10:93. 10.1186/s13023-015-0310-1) PMC4524368 26238698

[5] Watts SW Morrison SF Davis RP Barman SM . Serotonin and blood pressure regulation. Pharmacol Rev. 2012;64(2):359 388. 10.1124/pr.111.004697) 22407614 PMC3310484

[6] Boudreau DM Daling JR Malone KE Gardner JS Blough DK Heckbert SR . A validation study of patient interview data and pharmacy records for antihypertensive, statin, and antidepressant medication use among older women. Am J Epidemiol. 2004;159(3):308 317. 10.1093/aje/kwh038) 14742292

[7] Brody DJ Gu Q . Antidepressant use among adults: United States, 2015-2018. NCHS Data Brief. 2020;377(377):1 8. 33054926

